# Tumor mica status predicts the efficacy of immunotherapy with cytokine-induced killer cells for patients with gastric cancer

**DOI:** 10.1186/2051-1426-3-S2-P61

**Published:** 2015-11-04

**Authors:** Yu Chen, Zhi-feng Zhou, Wei-feng Zhu, Gang Chen, Yi Shi, Wan-song Lin, Zeng-qing Guo, Yun-bin Ye

**Affiliations:** 1 Fujian Provincial Cancer Hospital, Fuzhou, China

## Background

Accumulating evidence has demonstrated that cytokine-induced killer (CIK) cell immunotherapy may improve outcomes when used as an adjuvant to current standard treatment. Previous studies showed that cell signaling through MHC I-related Chain A (MICA)-Natural killer group 2, member D (NKG2D) results in CIK cells activation leading to cytolytic activities against tumor cells. In this study, we determine the relationship between the expression of MICA in gastric cancer tumors after D2 gastrectomy and the clinical outcome of a CIK containing adjuvant therapy.

## Methods

From January 2009 to March 2012, ninety-five consecutive patients with gastric cancer after D2 gastrectomy who received adjuvant chemotherapy combined with CIK cell therapy were enrolled (Table 1). The MICA expression of their tumors was determined by immunohistochemistry (IHC). The IHC score of was obtained by adding the intensity and percentage scores.

**Table 1:** 

Variable		N	m DFS	p-value	m OS	p-value
**Sex**	Male	66	42.0	0.373	44.0	0.229
	Female	29	42.0		50.0	

**Age**	<65	67	41.0	0.588	48.0	0.464
	≥65	28	43.0		43.0	

**Histological grade**	G1-G2	48	43.0	0.480	48.0	0.556
	G3-G4	47	31.0		43.0	

**Stage**	II	44	50.0	**0.001**	51.0	**0.006**
	III	51	36.0		41.0	

**Adjuvant Chemotherapy**	Xelox, Folfox4	57	41.0	0.250	43.0	0.257
	PF	38	42.0		46.0	

**CIK cycles**	<5	54	40.0	**0.046**	42.0	0.075
	≥5	41	48.0		50.0	

**MICA status**	High	38	46.0	**0.027**	48.0	**0.031**
	Low	57	41.0		42.0	

## Results

**Figure 1:**
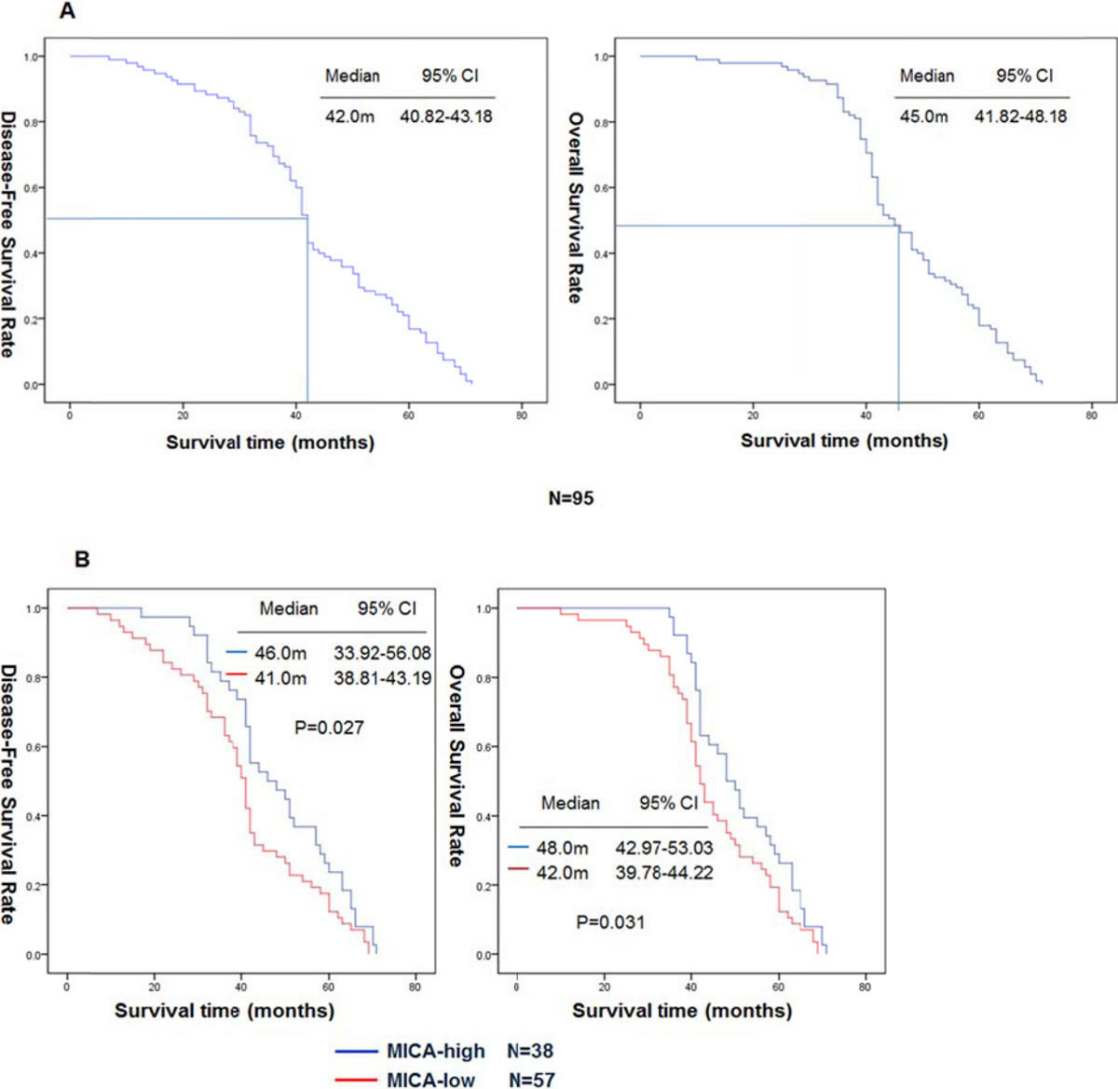


**Figure 2:**
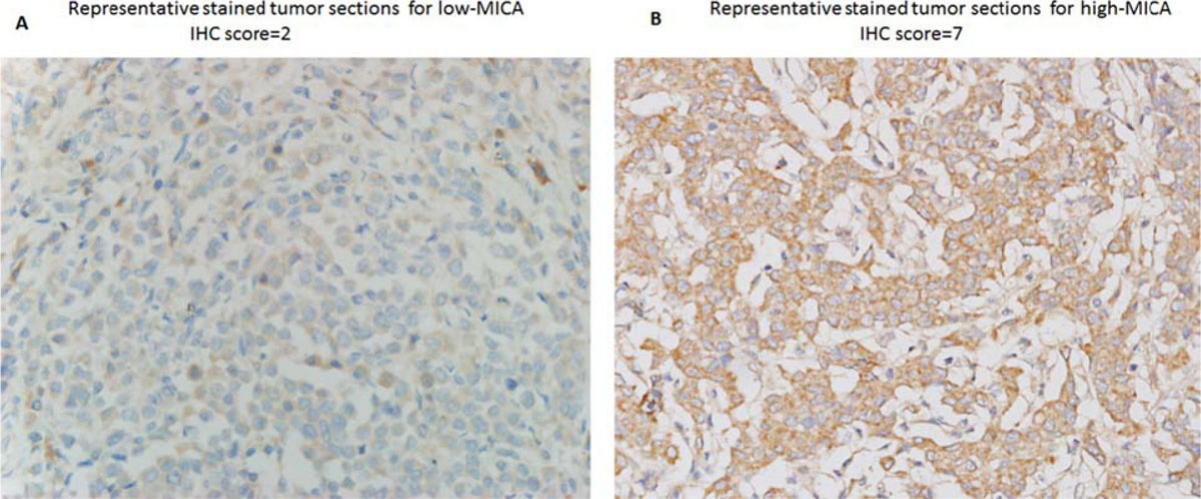


The MICA protein was detected mainly at the cell membrane and in the cytoplasm (Fig.1). High-expression of MICA protein, with IHC scores of 5-7, was documented in 38 of 95 tumor samples (40.0%). The MICA status was significantly association with the age and stage, p=0.008 and p=0.023, respectively (Table 2). Phenotypic analysis of NKG2D on *in vitro* expanded CIK cells showed that the percentage of NKG2D+ in CD3+/CD56+, CD3-/CD56+, and CD3+/CD8+ cells populations were 97.2±1.4%, 97.9±1.8%, and 95.6±2.1%, respectively. For the 95 patients, the median DFS was 42.0 months, 95% CI = 40.82-43.18 months, and median OS was 45.0 months, 95% CI = 41.82-48.18 months, the 3-year and 4-year DFS rates were 70.5% and 34.7%, respectively, and the 3-year and 4-year OS rates were 82.1% and 49.5%, respectively (Fig. 2A). For patient with high MICA expressing tumors the median DFS and OS were longer than for the patients with tumors with low expression of MICA; 46.0 months vs. 41.0 months (p=0.027), and 48.0 months vs. 42.0 months (p=0.031), respectively(Fig. 2B, Table 3). In a multivariate analysis, stage and MICA expression were independent prognostic factors for DFS and OS (Table 4).

**Table 2:** 

Characteristics		**Total95**	**MICA highN=38**	**MICA lowN=57**	p-value
**Sex**	Male	66	25	41	0.524
	Female	29	13	16	

**Age**	<65	67	21	46	0.008
	≥65	28	17	11	

**Histological grade**	G1-G2	48	23	25	0.111
	G3-G4	47	15	32	

**Stage**	II	44	23	21	0.023
	III	51	15	36	

**Adjuvant Chemotherapy**	Xelox, Folfox4	57	25	32	0.347
	PF	38	13	25	

**CIK cycles**	<5	54	18	36	0.128
	≥5	41	20	21	

**Table 3:** 

Characteristics		Number	Constituent ratio
**Sex**	Male	66	69.5%
	Female	29	30.5%

**Age**	<65	67	70.5%
	≥65	28	29.5%

**Histological grade**	G1-G2	48	50.5%
	G3-G4	47	49.5%

**Stage**	II	44	46.3%
	III	51	53.7%

**Adjuvant Chemotherapy**	Xelox, Folfox4	57	60.0%
	PF	38	40.0%

**CIK cycles**	<5	54	56.9%
	≥5	41	43.2%

**Table 4:** 

Variable	DFS			OS		
	P value	Hazard ratio	95%Ci	P value	Hazard ratio	95%Ci
**Stage**	0.001	1.915	1.270-2.886	0.010	1.713	1.138-2.577
**MICA**	0.035	1.578	1.033-2.409	0.040	1.557	1.020-2.376

## Conclusion

Our findings show that adjuvant chemotherapy plus CIK therapy treatment is a promising modality for treating gastric cancer patients after D2 gastrectomy. Especially, those who have tumors with high-expression of MICA were more likely to benefit from such a treatment strategy. Subsequent studies in clinical trial cohorts will be required to confirm the clinical utility of these markers.

